# Examining recovery and mental health service satisfaction among young immigrant Muslim women with mental distress in Quebec

**DOI:** 10.1186/s12888-024-05940-8

**Published:** 2024-07-02

**Authors:** Malka Reich, G. Eric Jarvis, Rob Whitley

**Affiliations:** 1 Department of Psychiatry, McGill University, Montreal, QC Canada; 2 Division of Social & Transcultural Psychiatry, McGill University, Montreal, QC Canada; 3 Douglas Mental Health University Institute, Verdun, QC Canada

**Keywords:** Religion, Mental health, Islam, Muslim, Immigrants, Stigma, Service utilization

## Abstract

**Objective:**

The overall aim of this study was to understand the experiences and perspectives of immigrant Muslim women in Quebec living with mental illness, who have recently used formal mental health services such as an accredited therapist, psychologist, or clinician. Specific objectives included (i) eliciting and examining their self-identified barriers and facilitators to recovery; (ii) exploring links between religion and mental health; and (iii) self-reported satisfaction with mental health services received.

**Methods:**

We adopted a qualitative approach, facilitating the prioritization of participant perspectives. This involved semi-structured interviews with 20 women who (i) identified as Muslim; (ii) had used mental health services in the last three years; and (iii) were 18 + years of age. Interviews were transcribed and analyzed using thematic analysis techniques.

**Results:**

Three prominent themes emerged from the analysis. These themes were (i) stigma and misunderstandings in families (especially parents) and sometimes in the ethno-religious community, both acting as barriers to health service utilization and recovery; (ii) frustrating clinical experiences within formal mental health care settings, in particular a perceived lack of cultural and religious competence, which negatively affected service utilization and the development of a therapeutic alliance; and (iii) deeply-held religious beliefs, practices and trust in God imparting a rhythm, purpose and meaning, which were strong facilitators to recovery.

**Conclusions and implications for practice:**

These findings suggest that recovery from mental illness can be advanced by a three-pronged approach in this population. First, anti-stigma mental health literacy interventions could be held in collaboration with Muslim community groups. Second, there is a need for further religious and cultural competence interventions, resources and trainings for mental health professionals working with Muslims. Third, self-care resources should be developed that harness aspects of religious practices that can give structure, meaning, purpose and hope. All this could ultimately foster recovery in this population.

**Supplementary Information:**

The online version contains supplementary material available at 10.1186/s12888-024-05940-8.

## Introduction

Like many G8 nations, the Canadian population is becoming increasingly diverse, primarily fueled by high levels of immigration. This heightened diversity has prompted Canadian research focusing on immigrant mental health. One concept that has been extensively documented in the literature is known as the “Healthy Immigrant Effect” which indicates that recent immigrants tend to be mentally healthier than the Canadian-born population, due to many strengths within immigrant individuals and communities [1, 2].

One well-researched topic relevant to immigrant mental health is religiosity, given high rates of religious involvement in immigrant groups [3, 4]. Research suggests that religiosity is a protective factor for mental health associated with greater psychological well-being, as well as lower rates of depression, anxiety, substance use and suicide [5–8].

Relatedly, other research indicates that religiosity can foster recovery from mental illness by acting as a source of strength, coping and resilience that can provide community, meaning and hope [9–11]. Religious practices that have been identified as helpful in this regard include prayer [12, 13], attending a place of worship [14–16] and consultation of sacred texts [17–19].

In acknowledging the benefits of religious practice on mental health prevention and recovery, it is important to note potential harmful aspects of religion on mental health. Some research suggests that certain religious doctrines such as a focus on the concept of sin or concerns about hell may induce excessive feelings of guilt, fear, shame, and self-loathing, potentially worsening mental health conditions [20]. Moreover, religious and cultural factors may contribute to the significant underutilization of mental health services by immigrants with mental distress compared to the native-born population; a phenomena repeatedly observed in studies in Canada as well as other Western countries [21, 22].

Such underutilization among immigrants per se has been attributed to various factors. First, immigrants have reported difficulties in finding culturally and religiously sensitive mental health service providers, with some even reporting experiences of discrimination within the health-care system, leading them to avoid or discontinue formal mental health care, [23–26]. Second, evidence suggests that immigrants may not consider emotional distress as a health issue, but instead as an existential or spiritual issue, leading them to consult resources outside the official health care system, such as religious healing or other culturally grounded means of help [27–30]. Third, there may be considerable stigma associated with mental illness and mental health service utilization among immigrant communities and families, leading individuals to avoid service for fear of negative judgement, reputational damage and other undesirable repercussions [23, 25, 31–33].

### Muslim immigrants

One cultural group whose mental health has been under-researched are Muslim immigrants to Canada [34]. This is a growing population, with recent statistics indicating that Muslims now constitute around 5% of the Canadian population [35].

Many of the aforementioned issues that have been detailed in the wider literature on immigrant mental health also apply to Muslim immigrants. For example, research indicates that there can be stigma within Muslim immigrant communities, with mental illness sometimes perceived as a consequence of insufficient faith or moral failing [31, 36]. Indeed, some research indicates that some Muslims may perceive mental illness as a punishment or test from God [31, 37], or as a result of supernatural causes such as Jinn, sometimes known as the “evil eye” or “spirit possession” [31, 38, 39].

Such beliefs can contribute to lower rates of formal mental health service utilization among Muslim immigrants, which in turn can hinder recovery, although evidence suggests they may be seeking help in different ways because of differing conceptualizations of emotional distress [34]. For example, several studies indicate that Arab immigrants in Western countries frequently attribute mental health problems to supernatural and religious factors and may prefer to seek help from religious healers rather than clinicians or therapists [40–42].

Other research, however, reveals that there is heterogeneity in mental health attitudes and beliefs among Muslim immigrants. For example, Muslims with high educational attainment and/or from the Middle East and North Africa more likely to use mental health services compared to those with less education, or from south or central Asia [43–45]. In other words, the literature suggests many nuances vis-à-vis mental health service utilization, recovery and stigma among Muslim immigrants, meaning a need for focused research on Muslim immigrant sub-populations.

One sub-population that has been under-researched are younger female Muslim immigrants with mental distress in Canada. Such women may experience a triple jeopardy in the host country on account of their gender, religion, and immigrant status, leading to experiences of discrimination which can contribute to poor mental health [46–48]. This may be particularly intense among veiled Muslim women, who may be vulnerable to religious discrimination given that the veil marks them as practicing Muslims [49, 50].

Moreover, research indicates that young Muslim women may experience intra-psychic, familial and cultural tensions regarding acculturation to the host culture. For example choices regarding attire, career, romantic partners, and social life may be considered undesirable by some family members and other members of the Muslim community, and may also incur some internal shame or guilt. All of this can cause psychosocial stress and mental distress [49].

Given this situation, the overall aim of this study was to understand the experiences and perspectives of immigrant Muslim women in Quebec living with mental illness, who have recently used mental health services, prioritizing their own perceptions. Specific objectives included (i) eliciting and examining their self-identified barriers and facilitators to recovery; (ii) exploring links between religion and mental health; and (iii) self-reported satisfaction with mental health services received.

## Methods

Given the lack of literature on the topic, we adopted a qualitative approach, aiming to conduct in-depth qualitative interviews with Muslims with mental health issues living in Quebec, Canada who had used mental health services. Precise inclusion criteria were as follows (i) aged 18+; (ii) of Middle Eastern or African descent, as these are the most populous Muslim groups in Quebec; (iii) had seen a therapist, clinician or doctor for mental health reasons in the public or private sector in the last three years, including those based at clinics or at other community settings; and (iv) able to speak English or French.

### Procedures

All participants took part in a semi-structured interview at a time and place of their choosing. Participants also chose their preferred language for their interview (either English or French). Interviews lasted between 45 and 90 min and were conducted by the first author (with the exception of one interview), who is a practicing veiled Muslim woman (see below). Interviews followed a topic guide aiming to elicit perceptions and experiences related to recovery, religiosity, and health service experience. This topic guide is included as a supplemental file. Questions on the topic guide included ‘tell me about your religion, what role does it play in your life?’, ‘you have recently seen a mental health professional. Tell me about that. How has that been going?’ and ‘what measures have you taken to aid your recovery?’ Participants were compensated with a $50 gift card for their time. All interviews were audio-recorded and transcribed verbatim into Word documents. The research protocol was reviewed and approved by the Research Ethics Board (REB) of the Centre intégré universitaire de santé et de services sociaux (CIUSSS) de l’Ouest-de-l’Île-de-Montréal –Mental Health (protocol number: IUSMD 21–30) and all participants gave written informed consent. All data were collected between September 2022 and April 2023.

### Researcher positionality

The first author was born in Montreal, Canada and is a practicing veiled Muslim woman, speaking several different community languages. This not only means that she is an insider to the community with knowledge of Islamic theology and concepts, but also has lived experience as a veiled Muslim women in Quebec. This author conducted all bar one of the interviews. This may have facilitated interviewer-interviewee rapport, and contributed to openness from participants, all of whom were Muslim women. The two other authors are not Muslims; however they are both experts in the sub-field of religion and mental health, having both spent several decades researching the experience of religious minorities. This balance in the research team may have achieved the desired middle ground between insider-outsider status; an advantageous position when conducting research with minority groups [51].

### Recruitment

We made strident efforts to publicise the study in different on-line forums and physical locations. To begin, flyers advertising the study and soliciting participation were created and distributed in various targeted locations. First, the flyers were posted on several relevant Facebook groups. Second, the research team is based at a hospital within a wider network of clinics and other health care services. We posted the flyer in waiting rooms across this network, and also worked with clinical collaborators, who agreed to distribute the flyer to any patients who might meet the inclusion criteria. Third, we contacted mosques and Muslim religious organizations across the Greater Montreal Area, offering to meet and discuss the research. Two of them disseminated information about the study to their members. Finally, we engaged in snowball sampling, asking participants to spread the word about the study to individuals in their social network.

### Participants

The above recruitment strategy led to 23 participants meeting the inclusion criteria and partaking in an in-depth qualitative interview. Only three of these participants were male, and information revealed during interviews indicated that these three participants did not truly meet the inclusion criteria. Consequently, we dropped the men from the analysis and focused on the women, consistent with the literature presented in the introduction indicating their specific issues.

As such, our final sample comprised 20 women between the ages of 18 to 42 (mean = 28), with 65% of participants below the age of 30, and 85% below the age of 35. Two were married, three were divorced, and 15 were single. The sample was relatively educated compared to the general population, with all but one participant holding or pursuing a bachelor’s degree. This included four participants with a post-graduate degree. Among the participants, 11 were still students at the time of the interview. All but three participants were first-generation immigrants, with an average of 8.4 years in Canada. The three second generation participants born in Canada had parents who had immigrated to Canada. Participants were from a variety of countries including (inter alia) Algeria, Morocco, Lebanon, Syria, and Turkey. Participants reported experiencing a variety of mental disorders, most commonly anxiety and depression.

### Analysis

The transcribed interviews were imported into MaxQDA for analysis. Analysis was propelled by the thematic analysis approach outlined by Braun and Clark [52], an iterative process of identifying patterns and themes within a dataset through continuous engagement with the raw data. At the initial stages of analysis, the first and last author immersed themselves in the data through reading and rereading transcripts. The first author then created a preliminary coding tree with codes created to reflect prominent and repeated material appearing in the interview. This tree was revised by the last author during initial coding to ensure it captured dominant patterns and themes. The first author then coded all the interview transcripts using the finalized coding tree. On completion of this task, the research team met to create themes from the codes, which sometimes involved merging or collapsing codes into a wider theme. Finally, the most prominent themes were named and defined as faithful reflections of repeated and prominent experiences and perceptions that appeared throughout the dataset and presented below in the results.

### Rigour

Several steps were taken to improve rigour in this study. First all interviews were conducted by a female interviewer (all bar one by the first author) who is a veiled Muslim woman. This may have increased rapport, openness, and honesty among participants. Second, the data was subject to multiple coding by the first and last author, both coming to similar conclusions about prominent themes. Third, the data was deliberately re-examined during the ‘revise and resubmit’ stage by the authors, to specifically assess for the presence or absence of other themes raised as potentially important by one of the reviewers. This re-examination confirmed that the research team had not missed any potentially important themes. All these methods have been identified as enhancing rigour in qualitative research [53].

## Results

Three prominent themes emerged from the analysis. These are (i) stigma and misunderstandings in families and the ethno-religious community (ii) misunderstandings by therapists and issues around cultural safety and (iii) religious beliefs, practices, and trust in God. Themes one and two were primarily barriers to recovery and health care utilization, while the third theme was mainly reported as a facilitator of recovery. All quotes are verbatim, except for minor changes to demographic information to protect participant anonymity. Excerpts from French interviews were translated into English by the authors.

### Stigma and misunderstandings in families and the ethno-religious community

Many participants noted that their parents typically held stigmatizing attitudes and negative beliefs about mental illness and mental health service utilization. For example, one participant stated that her parents “absolutely refused” (M02F) to get her a therapist, noting that “they think maybe she’s white and she’s putting in, like, Canadian thoughts into my head, and that’s why she’s supporting me in, in terms of my life decisions. So, it’s discouraged in that way” (M20F).

For those who felt safe enough to confide in their parents, many reported that their struggles with mental health were minimized, dismissed, and invalidated. For example, participants reported hearing statements like “everyone feels depressed”, “everyone went through their own things”, and “we can inflict depression onto ourselves” from their family members. This is illustrated in the quote below, from a 36-year-old Moroccan immigrant:

In- in my family, and even in society, it- it’s usually the belief that- they tend to be very dismissive about all, like, mental health conditions if- like, if you- say, for instance, you were depressed, “Oh, you’re just ungrateful.” Or, if you’re- like, if you’re having- I don’t know. If you’re obsessed with certain thought or you can’t focus or you limit- if you have a serious, like, psychotic problem, they would be like, “Oh, maybe you’re possessed. Or maybe you’re-”, you know? They- they tend to, like, be a little bit dismissive. (M01F)

Indeed, participants often reported that parents tended to express literal incomprehension that their offspring could have a mental illness, noting that such health complaints were perceived as a form of ingratitude by parents, who sometimes drew comparisons with the harshness of life in their countries of origin compared to the peace and order of Canada. For example, a Canadian born participant describes a conversation with her mother, a refugee from Afghanistan below:

And I think my mom, I was telling her that I wasn’t doing really well and I didn’t really like one of the- like how she reacted one time. She’s like, “why are you depressed!?” Like, “I had a reason to be depressed! I did this in Afghanistan. I did that.” So she kind of minimized my issues (M19F).

Furthermore, some participants noted that their family members, as well as other members of their ethno-religious communities, sometimes equated any mental illness with being “crazy”. A 33-year-old Iraqi Canadian dealing with anxiety and depression noted that mental health was stigmatized in her home, stating that “for Iraqis, or for my dad anyway, like, any kind of mental health issue is, ‘Oh, you’re crazy!’.” Another participant described a similar mentality:

You know- you still whisper about someone who has a mental illness. You still whisper about it. You don’t say it loud. You don’t- it’s bad. You know, it’s really bad. It’s- it’s- it’s crazy people who go there to the crazy hospice, you know. (M11F)

Interestingly, many participants noted that family or community members sometimes attributed any mental health issues to supernatural possession or insufficient religiosity. As previously mentioned, one participant noted that community members might say “Oh, maybe you’re possessed” in response to “psychotic problems”. Another participant recalled a conversation with her mother where she was told “You don’t need a therapist. You just read Qur’an” (M01F). Other participants reported being advised by family members to “go pray” or “read Qur’an” instead of seeking help from mental health professionals. All this is encapsulated in the quotation below:

Community. I feel like there’s a big, big, big taboo around mental health and especially when it comes to religion, you know, like people are like, oh, it’s just you have like low *imaan* (faith) and like, oh, you just like need to be closer to God (M13F).

In other words, families did not always provide a supportive environment for disclosure, and did not typically encourage or support service utilization. On the contrary, families could be a barrier to recovery, by invalidating the existence of the illness and minimizing its gravity. Knowledge of such parental beliefs meant that many participants decided not to discuss their mental health struggles with parents and maintained some level of secrecy regarding mental health service use. For example, a 21-year-old Middle Eastern immigrant described how she met with her therapist in secret and did not tell her parents because “they grew up in a different condition” and didn’t think “they would understand it” (M18F).

In fact, the stigmatizing attitudes and negative beliefs were often internalized by participants, leading to self-stigma, which could be a barrier to service utilization and recovery. This is illustrated by a participant who stated that “like in our culture it’s more like, why would you see a therapist and all that? And I kind of got into that mentality at some point and it was hard for me to reach out to get a therapist”. (M19F).

In sum, participants typically reported stigmatizing attitudes and negative beliefs towards mental health and mental health service utilization in their family unit and within the wider ethno-religious community. Nevertheless, a few participants also expressed that some family members were helpful in their recovery. For example, a 19-year-old Lebanese immigrant recounted how her mother helped access mental health services: “my mom was adamant on finding someone because she- she acknowledges the importance of, like, seeking a professional, even if it’s like little things that you can do, like studying or, you know, mental health” (M16F). Another participant noted that she continually confided in her mother, who was helpful in accessing services, as well as supporting her recovery:

“Yes. So I’m I’m very, very close to my mom. She’s pretty much aware of everything that’s going on in my life. And she is my first therapist to some extent. So she knew about the whole performance anxiety. She saw me having a panic attack in the middle of the night so many times she like saw that I was not happy, that I was not well. And she’s the one who actually suggested that I started seeking help for – from a therapist. (M13F).

### Misunderstandings by therapists and issues around cultural safety

An overwhelming majority of participants noted that there were problems in developing a positive therapeutic alliance with therapists and clinicians. In particular, they noted that the ethno-cultural background of therapists and clinicians normally played an important role in service satisfaction and recovery. Typically, participants noted that it was difficult to build an effective therapeutic alliance when there was an ethno-cultural mismatch, with one participant stating that “I sometimes feel like it’s harder to explain some feelings to therapists or psychologists from a different cultural and religious background than you, because coming from a certain religion, a certain culture, you learn to interpret things differently.” (M09F). These sentiments were echoed by a 27-year-old Syrian immigrant, stating that:

“Like, for example, now I am on a waiting list for a therapist, but I am 100% sure the therapist I’m gonna be assigned to will not understand many of the challenges I am going through. So, it’s- like, we’re not gonna be able to relate. If I want- if I want someone I can relate to, someone with the same cultural background, I have to pay.” (M03F).

Participants regularly stated that therapists from the majority culture had a limited understanding of the cultural and religious context of participants’ lives, especially regarding dynamics and values within Muslim families. For example, one participant stated that “she’s [the psychologist] Quebecoise, so, like, it’s kind of limited in that- in the sense that she doesn’t really know, culturally, what it’s like to be in an Arab family (chuckles) or a Muslim family”. (M05F). Another participant described an interaction commonly recounted by participants, namely therapists encouraging participants to exert autonomy and become independent from family ties that were perceived to restrict individual growth. Such advice was considered inappropriate:

“But, you know, when I was with like those Quebecer therapists, they just didn’t get that. They’re like, well, just do it like it’s fine. It’s your own life. And I was like, no, you don’t get it. I can’t. I can talk about it all I want, but I can’t actually do it. And so, yeah, so to me, it was kind of like that cultural clash, if you want. That their advice just didn’t always work with what I believed in or how things kind of like function in my family. And they weren’t really trying to adapt to it either. It was just like, well, no, you can do whatever you want. It’s like, no, I can’t, no.” (M13F).

Participants noted that this lack of knowledge meant that they had to act as community educators to clinicians, at some cost to their own well-being, as such exertions involved precious psychic energy. For example, a 23-year-old participant noted that “because it’s just like you- you- just have to like, waste so much time explaining to someone, like, something and stuff.” This attitude was shared by another participant who said that she “just didn’t feel like it was really efficient, and, quite honestly, at that time, I didn’t really have energy to go an extra mile to explain”.

Another issue raised by the participants was a mismatch between advice offered by the therapist and Islamic values held dear by the participant, which could negatively affect the therapeutic alliance. For example, one participant stated that “another thing I just remembered, because I was showing symptoms of depression and I was just like not into anything…and he said, ‘Oh, what if you remove your hijab, maybe that will make you feel better.’” (M19F) Another participant noted how a similar mismatch led her to seek new therapists who might have a better understanding of Islamic life, but to no avail, as stated below:

“One of the struggle is to find someone who will actually give you advice that is *halal* [permissible under Islamic laws] or that works with our religion. So I’ve never been able to umm… get that support, although I did have lots of therapists over- in the last uh… few years, I’ve had like over ten therapists easily” (M04F).

More than half of the participants stated that they would specifically prefer a Muslim therapist. For example, a 36-year-old Tunisian immigrant expressed that her “first reflex was to see a Muslim, um, therapist”. She explains: “I wanted someone who knows what I come from. Like, who wouldn’t- who would, like, give me advice that’s- would be in line with my values”. Despite preferring to have a Muslim therapist, only a few participants were able to find one. Among them, one Syrian immigrant, spoke of her positive experiences with a Muslim therapist she found online, after a history of negative experiences in local clinics, stating:

Yeah, actually, [the therapist]- like, I what I liked about her is that she was- her therapy was very Islam-oriented. So, she was always implementing hadith and *Ayat* (verses of the Qur’an) in her- in the way she was talking to me…she just understands where- where I’m coming from.

Another participant, a 20-year-old Syrian, expressed: “I definitely felt 100% more, like, comfortable with the Muslim, like telling her more about my parents because I knew she understood it. Umm… And I definitely would prefer like another like Muslim therapist.”

Interestingly, some participants reported positive experiences with non-Muslim therapists who followed another religion, or who came from cultures that shared similar family dynamics and values. For example, one participant stated “So, she’s Lebanese. She’s not Muslim. I think she’s Catholic, but she is- she understands a lot of the immigrant struggles or first-generation struggles, I should say.” (M16F). Another participant lauded her Greek immigrant therapist:

“She is a very like practicing Christian. So, you know, want it or not, our religions are still very similar in terms of like values. So that really helps because when I tell her about, let’s say whatever comes up that religion can be implicated in, then she will understand. And when it comes to, for example, that thing about moving out, well, she knows. Like Greeks they’re pretty much the same as Arabs when it comes to that. It’s very family oriented, very community oriented. So, she is able to advise me around those realities because she relates to them as well, which makes it easier for sure.” (M13F).

While the majority of participants reported a preference for cultural matching, others noted that this was not always the ideal situation for an individual. For example, one participant noted that cultural matching “has its pros and cons”, while another 32-year-old participant expressed a preference for a therapist from the majority culture, stating:

“I prefer to have a therapist from here because I’m integrating here in the adjustment period. So I need someone from here that can help me to adjust here because the therapist from the Middle East doesn’t know about here well and maybe cannot help me better for- for a better life here.” (M07F).

### Religious beliefs, practices and trust in God

The first two themes were principally barriers towards recovery and service utilization. However, the present theme is principally a facilitator towards recovery, related to the positive impact of religious practices and a meaningful relationship with God. Centrally, many participants described how such religious practices and religious faith helped them, especially during difficult times. This is illustrated by the participant below:

“So, I guess it’s like this with every religion. But for me, mostly, when I feel alone or- basically, it’s like a backbone for me. So, it gives me a sense of security, especially when I say prayers or when I’m- when I’m in a situation that I don’t know what to do or when I’m feeling alone. It’s- it’s kind of a hope, a sense of not being alone, a sense of home” (M23F).

The theme of religious faith giving security, foundation, direction and hope during difficult times reappeared throughout the dataset. For example, one participant stated that “I feel like if I didn’t have any kind of religion, then I’d be a little bit lost. But to have- it’s kind of, like, having a base for me. So that’s comforting…”. Additionally, many participants revealed that their religious faith and practices gave them other psychosocial benefits, including a deep sense of meaning and purpose, as evidenced by a 26-year-old Algerian participant:

Well, I consider my religion as… it’s…. Without that my life- I would be, I would be truly lost. My life would be disjointed. My life would be upside down and I wouldn’t really have a goal. And I wouldn’t really have any meaning in my life. I wouldn’t know what’s good and what’s bad. I wouldn’t know why, like, certain things happen in my life. In fact I would be lost, I would really be lost. And sometimes, I understand why people commit suicide because they have no goal, they have no meaning in their lives. (M14F)

In terms of religious practices, participants described “talking to God”, usually through prayer, as providing them with peace, structure, ease, assurance, as well as stress and anxiety relief. For example, one participant explained that prayer will “bring you more *Barakah (*blessings*)* in your life and in your mental health as well” (M19F). Others reported that abandoning prayer had a negative impact, with one participant stating “but I can feel like right away after like a month or two of not praying, that like my life is not going well. It’s going downhill” (M04F).

In conjunction with discussion about the positive benefits of prayer, almost all participants frequently noted the essential importance of a trusting relationship with God in their recovery journeys. For example, one participant stated that “I feel like without religion and this connection with God, I would have never been able to make it. And like, I think I would have actually killed myself”. Many participants describe being able to overcome hardships through God’s help. For example, one recounts: “No matter how low- how low I- I go, like, how bad things get, I know that God is there for me. I know that I can always, like, lean on him and pray for him” (M03F).

For many, this was rooted in a profound trust in God and his perceived plan. Participants often talked about “trusting the process”, “trusting God”, as well as the idea that “God knows what’s good for you”. Importantly, prayer and reliance on God were not typically seen as replacements for therapy, but as helpful facilitators of recovery that could amicably co-exist with therapy. For example, one participant stated that “I know Islam will also encourage, like, prayer for healing, but I don’t think it discourages good mental health…as far as I’m concerned, I feel like the faith actually promotes, like, peace and healing and all these positive things” (M20F). Similarly, another participant stated that:

“prayer, of course, or even talking to *Allah* when I was like sitting in any condition, not just at the prayer mat or in a specific situation, all the moments- like because I believe that *Allah* is the only friend I had, that that difficult time. And therapy was a complementary help” (M08F).

Another factor that was considered helpful for recovery was consultation of the Qur’an, Islam’s sacred text. Indeed, many participants discussed how listening to or reading the Qur’an in times of difficulty provided comfort and support. Some participants recalled specific verses that resonated with them, mainly verses which reinforced God’s presence, support and guidance in their lives. These included “Indeed my Lord is with me, and he will guide me” (Qur’an 26:62), “take one step towards me, I’ll take ten steps towards you. Walk towards me, I’ll run towards you” (Hadith Qudsi), “So be patient with your Lord’s decree, for you are truly under Our watchful eyes” (Quran 52:48), and “I entrust my affair unto God. Surely, Allah is watchful over His servants” (Qur’an 40:44).

Some of the above-described religious practices (e.g. prayer, trust in God, meditation on sacred texts) are common across monotheistic Abrahamic faiths. However, participants also detailed how concepts and precepts unique to Islam were helpful in their recovery. For example, many participants described finding solace by mediating on some of the 99 attributes/names of God, detailed in Islamic teachings, especially *Al Rahim* (The Most Merciful), but also *Al Rahman*, (the Most Gracious), *Al Salam* (Giver of Peace), *Al Wadood* (The Loving One), and *Al Muhaymin* (The Protector).

Similarly, the concept of *Mektoub* (destiny) was referred to by participants. This concept is part of Islamic teaching declaring that fates are predetermined, and that God had already written down everything that will happen during every human’s lifetime before they were born [54]. For some participants this concept was very helpful, with one stating that: “The whole concept of *Mektoub*. […] I feel like this is the best way of kind of like, to accept things, so, you know, just be like it’s *Mektoub*. There’s a reason for it. I don’t know what yet, but I will know it eventually.” (M13F).

Despite the numerous therapeutic aspects of religiosity discussed above, a few participants mentioned that some factors related to religion could act as a barrier to recovery. For example, some participants felt that they were falling short of God’s expectations due to a perceived lack of progress and devotion in their lives because of mental illness, leading to feelings of guilt and worthlessness. Indeed, participants alternately described feeling “not good enough”, like a “bad Muslim”, or like they “could or should do better” for their perceived inactivity. One participant illustrated this point by explaining that: “It’s a little hard ’cause when I think of God, sometimes I just feel really guilty and I feel like a piece of shit (laughs) and just not good enough.” (M05F).

Other participants described a “vicious cycle” whereby they felt too depressed to complete religious tasks, in turn plaguing them with intense feelings of guilt that worsened the initial depression. A common religious activity that elicited feelings of guilt when abandoned was prayer, with one participant explaining:

But sometimes it- it gets, like, almost hard where, like- like struggling with mental illness and, like, when you hate yourself for not doing something, it’s, like, almost invalidating to, like, restart the thing again. If that makes sense. So, it would be like- like I would stop praying or something and then have this guilt about it, but then, like, not do anything about it, but then, like, the guilt would just keep growing. And so, it’s just, like, a never-ending cycle where, like, I don’t know what to do with it, basically. (M21F).

## Discussion

There are three key findings of this study. First, the study revealed that participants perceived high levels of misunderstanding and stigmatizing attitudes towards mental illness in their close family, as well as in their wider community. Many participants reported that their parents and other family members often attributed their mental health issues to insufficient religiosity. These types of misunderstandings are not unusual, with previous research indicating that it is common in Arab Muslim culture to believe that mental illness is caused by insufficient faith [31, 55]. In this mainly younger sample, the views of parents and older Muslim people were often singled-out as either dismissive, stigmatizing or invalidating. This inhibited health service use and disclosure, leading to secrecy and silence about mental health in some families.

This overlaps with previous research indicating that social stigma surrounding mental health issues in Arabs and Muslims appears to be common and impedes service utilization and help-seeking behaviors [31]. That said, it is important to state that mental health stigma exists in virtually all cultures and faith groups, however it can manifest somewhat differently according to the precise community [56]. Knowing the different manifestations can inform anti-stigma strategies as discussed later in the section on implications.

Second, participants reported that therapists and clinicians typically lacked cultural and religious competence and understandings, leading to a largely negative experience with clinical care. Many examples were given, but a prominent and repeated example related to issues surrounding the family. Participants reported that therapists implicitly valued individual autonomy and explicitly encouraged personal independence from family; values that are strong and normative in Euro-Canadian culture. However, this advice was given without due consideration of norms, customs and values commonly held by Muslim families, including the importance of family harmony, an abiding sense of familial obligation, and a strong expectation of intergenerational support. To participants, such clinical advice seemed trite and ignorant, contributing to negative experience of care. This led many participants to avoid or drop out of formal mental health care, or at least contributed to a dissatisfying experience and a constant search for new therapists.

This negative experience is consistent with previous research indicating that immigrants often underutilize mental health services due to a weak therapeutic alliance where misunderstandings by clinicians are commonly encountered [25, 26]. Indeed, most participants stated that they preferred therapists who came from a similar cultural or religious background. Interestingly, other research indicates that matching minority clients with clinicians from the same background can increase mental health service utilization, therapeutic alliance, and service satisfaction [57–59]. However, such matching may not always be possible, given that the supply of mental health clinicians from religious and cultural minorities may not always meet the demand. All this indicates the need for wider training in cultural and religious issues for all clinicians, further discussed below. Another solution is greater use of on-line mental health counselling, which is now commonplace after the COVID-19 pandemic, increasing the possibilities of culture or faith matching for individuals seeking minority therapists, without geographical limits.

The third key finding is that participants typically reported that aspects of their religious beliefs and practices helped foster recovery from mental illness. Indeed, frequent prayer, recitation of the Qur’an and other religious practices helped anchor and stabilize individuals in mental distress, giving a sense of meaning, purpose and hope. In sum, this finding is consistent with the large corpus of research discussed in the introduction indicating that religiously is positively associated with recovery [60–62]. Furthermore, participants reported that therapy was especially effective when their religious beliefs were properly considered and incorporated into treatment, overlapping with other literature on the importance of religious competence in the clinic [60, 63].

All this implies the need for innovative approaches to the integration of therapy and religion to better serve Muslims with mental illness. One approach could involve mental health professionals training Imams and other leaders at Islamic Centres and Mosques in Mental Health First Aid and psychoeducational interventions, so that these faith leaders can in turn support their congregants. Such bridging and collaborative initiatives have already proven to be successful in some settings in Canada and the USA, with several partnerships created that deepen reciprocal knowledge between mental health professionals and religious leaders [64]. In fact, there are already Mosques and Centres in Canada and elsewhere offering different forms of counselling for Muslims with mental health issues, both in-person and on-line, however these are not well-known nor well-funded. As such, further partnerships between official mental health services and religious organizations could be explored to solidify links and mutual learning opportunities.

Interestingly, not one participant expressed a belief that religious healing is the only route to recovery. Likewise, not one participant attributed their mental distress to supernatural factors. As such, the findings diverge from some other studies showing that some Muslims perceive mental illness as a test or punishment from God [31, 37], or as a result of Jinn, the “evil eye” or “spirit possession” [31, 38, 39]. This may be because other samples include a wider range of participants in terms of educational attainment, age and region of origin, while the present sample was characterized by higher levels of educational attainment, a younger age and Middle East or African region.

### Limitations of the study

There are several limitations to this study. First, the relative homogeneity of the sample is one of the limitations of the study, as the sample was wholly female, and skewed towards younger highly educated people from the Middle East. Different results may emerge from studies of older people, men, people with lower educational attainment and from other parts of the Islamic world. These should be an area of future research. Second, the project was carried out in a single province of Canada, Quebec, which is a distinct society that officially embraces secularism through various laws and policies. As such, caution is advised before extrapolating the results to other Muslim sub-populations and Muslims living elsewhere. Third, inclusion criteria included the ability to speak English or French (the two official languages of Canada), which excludes immigrants who are only able to speak their heritage language. Such immigrants may have a more difficult experience and may face even more intense issues with health service utilization and satisfaction; however they were not included in the study. Again, this could have biased the results. Fourth, the research was conducted by a member of the community, however it was not a community-based research project done in collaboration with Mosques or other Muslim community groups. Fifth, the participants frequently discussed their own parents’ attitudes and beliefs in the interviews, for example parental apprehension about their daughters using mental health services. However, we did not collect any data from parents, meaning we are lacking their unmediated perspectives and perceptions about the issues discussed. Further research is necessary to understand Muslim parents’ knowledge, beliefs, behaviours, and attitudes towards mental health issues, especially pertaining to their sons and daughters.

### Implications for therapists, religious leaders, and future research

These findings suggest that recovery from mental illness can be advanced by a three-pronged approach in this population, with specific implications for therapists, clinicians as well as religious community leaders. Any new action must be accompanied by appropriate research and evaluation, described in detail below.

First, tailored mental health literacy interventions could be conducted in collaboration with Muslim community groups, for example religious congregations, to educate community members about the reality of mental health issues, services available and pathways to recovery, especially targeting older people and parents. These must be culturally and religiously appropriate, for example by incorporating some of the concepts mentioned as helpful for recovery by participants, including specific verses from the Qu’ran and the 99 names/attributes of God. Moreover, such interventions could be delivered by specially trained community members, with religious and community leaders involved in the creation of tailored content. This could build on knowledge accrued by existing anti-stigma and psychoeducational programs focused on addictions that have been successfully implemented by Muslim mental health professionals in Mosque settings in Canada [65]. Such bottom-up interventions could be tested for acceptability, feasibility, uptake, and impact, and if successful scaled-up elsewhere.

Second, there is a need for further religious competence interventions, resources and trainings for mental health professionals working with members of the Muslim community, with an emphasis on understanding and harnessing religiosity as a resource for recovery. Again, this could involve new tailor-made trainings, developed by mental health clinicians working in tandem with religious leaders and authorities. Such trainings could include first-person testimonies by Muslims with lived experience of mental illness (either in-person or via video capsules), which has proven effective in reducing stigma and raising awareness of common mental health issues faced in other contexts [66]. Furthermore, these trainings can also discuss how clinicians can culturally-adapt evidence-based clinical tools such as cognitive behavioural therapy so that they are more appropriate and relevant for Muslim patients; an emerging yet promising practice in psychotherapy [67]. This must be accompanied by parallel research which examines impact on knowledge, beliefs, behaviours, and attitudes of clinicians participating in the training, and ultimately impact on patient engagement with services and recovery outcomes.

Third, self-care and self-management resources can be developed that encourage regular engagement in religious practices as a route to recovery, giving structure, meaning, purpose and hope to Muslims with mental illness. Such self-help resources have become common place in the field of psychiatry for many different disorders (though without any religious content), and a recent systematic review and meta-analyses indicated that they can be effective in promoting recovery and facilitating other desirable psychosocial outcomes [68]. However, to our knowledge, these resources are rarely tailored to religious populations or incorporate religious themes. This implies the need for the creation and evaluation of such resources, which could eventually be made available on-line and for therapists (Muslim and non-Muslim alike) to distribute to their clientele.

## Conclusions

Taken together, the findings reveal that younger Muslim women with mental distress typically feel misunderstood by important sectors of society, including their parents, their wider social circle and their mental health caregivers. This can leave them frustrated and isolated. However many find solace in their religious beliefs and practices, which provide a balm to their mental distress. Further action is necessary to reduce stigma, improve clinical care and foster recovery in this population.

### Electronic supplementary material

Below is the link to the electronic supplementary material.

Supplementary Material 1

## Data Availability

Anonymized excerpts of the transcripts relevant to the study are presented within the paper. Full transcripts cannot be shared publicly because they include massive quantities of identifying and highly-personal information. The Research Ethics Board (REB) approval was given contingent upon the promise that data would only be shared among the actual research team.
