# Supplementary material for: Examining recovery and mental health service satisfaction among young immigrant Muslim women with mental distress in Quebec
Source: BMC Psychiatry. 2024 Jul 2;24:483. doi: 10.1186/s12888-024-05940-8 (PMC11221043; doi:10.1186/s12888-024-05940-8)
Supplement: Supplementary file 1 — Supplementary Material 1 [file 12888_2024_5940_MOESM1_ESM.pdf]

Interview script

1. First off, can you tell me a bit about yourself?
2. Where are you from?
3. (IF APPLICABLE) how long have you lived in Canada?
4. (IF APPLICABLE) Tell me about life in your country of origin, what was it like?
5. (IF APPLICABLE) Please share your immigration story with me. Why and how did you end up here?
6. (IF APPLICABLE) What has it been like for you living in Canada and Quebec since?

RELIGIOSITY

7. Tell me about your religion
8. What role does it play in your life?
9. How involved are you in your religious community?
10. Tell me about your regular religious activities (e.g. prayer, attendance, bible study etc.) [include prompts (i) activities pursued alone; (ii) with family; (iii) in a congregation]
11. How do you feel your religion is perceived here in Canada and Quebec? [prompt for any positive or negative individual experiences]

EXPLANATORY MODELS AND KABB

12. You have recently seen a mental health professional. Tell me about that. How has that been going? [prompt for levels of satisfaction, engagement, religious competence etc.)
13. What do you think caused the issues leading you to see a mental health professional?
14. Have you discussed these issues with any religious leaders, ministers or healers? If so, please tell me about that. How has that been going?
15. What other measures have you taken to address these issues? [prompt for (i) health care e.g. medication, therapy, etc.; (ii) religion, e.g. prayer, faith healing etc.; (iii) other]
16. What impact have these measures had on your recovery? [prompt for each separate measure mentioned above]

CONCLUSION

17. Thank you for your time, is there anything else you would like to add?
